# Regulatory role of *Mycobacterium tuberculosis* MtrA on dormancy/resuscitation revealed by a novel target gene-mining strategy

**DOI:** 10.3389/fmicb.2024.1415554

**Published:** 2024-06-17

**Authors:** Xiang Fu, Xiaoyu Wan, Aadil Ahmed Memon, Xiao-Yong Fan, Qiuhong Sun, Haifeng Chen, Yufeng Yao, Zixin Deng, Jian Ma, Wei Ma

**Affiliations:** ^1^State Key Laboratory of Microbial Metabolism, School of Life Sciences and Biotechnology, Shanghai Jiao Tong University, Shanghai, China; ^2^Department of Respiratory and Critical Care Medicine, Shanghai Pulmonary Hospital, Shanghai, China; ^3^Shanghai Public Health Clinical Center, Shanghai Institute of Infectious Diseases and Biosecurity, Fudan University, Shanghai, China; ^4^Department of Laboratory Medicine, Shanghai East Hospital, Tongji University School of Medicine, Shanghai, China

**Keywords:** *Mycobacterium tuberculosis*, MtrA, regulatory network, dormancy, resuscitation, persistence

## Abstract

**Introduction:**

The unique dormancy of *Mycobacterium tuberculosis* plays a significant role in the major clinical treatment challenge of tuberculosis, such as its long treatment cycle, antibiotic resistance, immune escape, and high latent infection rate.

**Methods:**

To determine the function of MtrA, the only essential response regulator, one strategy was developed to establish its regulatory network according to high-quality genome-wide binding sites.

**Results and discussion:**

The complex modulation mechanisms were implied by the strong bias distribution of MtrA binding sites in the noncoding regions, and 32.7% of the binding sites were located inside the target genes. The functions of 288 potential MtrA target genes predicted according to 294 confirmed binding sites were highly diverse, and DNA replication and damage repair, lipid metabolism, cell wall component biosynthesis, cell wall assembly, and cell division were the predominant pathways. Among the 53 pathways shared between dormancy/resuscitation and persistence, which accounted for 81.5% and 93.0% of the total number of pathways, respectively, MtrA regulatory genes were identified not only in 73.6% of their mutual pathways, but also in 75.4% of the pathways related to dormancy/resuscitation and persistence respectively. These results suggested the pivotal roles of MtrA in regulating dormancy/resuscitation and the apparent relationship between dormancy/resuscitation and persistence. Furthermore, the finding that 32.6% of the MtrA regulons were essential *in vivo* and/or *in vitro* for *M. tuberculosis* provided new insight into its indispensability. The findings mentioned above indicated that MtrA is a novel promising therapeutic target for tuberculosis treatment since the crucial function of MtrA may be a point of weakness for *M. tuberculosis*.

## 1 Introduction

Tuberculosis (TB) caused by *Mycobacterium tuberculosis* infection remains a serious worldwide health concern in terms of infection incidence and associated mortality (Kone et al., 2022), despite the significant improvement in the situation; for example, the global mortality of TB considerably reduced by 47% from 1990 to 2015 through the global efforts of many countries, including China (Wang et al., 2014; World Health Organization, 2015). However, the incidence of tuberculosis is on the rise again; for instance, its global incidence rate in 2021 increased by 3.6% compared with that in 2020 (Ding et al., 2020; World Health Organization, 2022). Although approximately 70% of new tuberculosis cases are diagnosed predominantly in developing countries, especially in regions such as Southeast Asia and Africa, which have become heavy burdens for these countries (World Health Organization, 2022), the incidence rates in developed nations are increasing significantly as well, especially for vulnerable populations, such as children and AIDS patients (Dheda et al., 2016; Tomà et al., 2017).

Since the metabolism of dormant bacilli is considerably suppressed (Shleeva et al., 2015; Jain et al., 2016), DNA replication (Gopinath et al., 2015) and cell division (Muñoz-Elías and McKinney, 2006; Iona et al., 2016) are arrested, and even cell wall composition and structures are altered (Bacon et al., 2014), dormancy/resuscitation could significantly improve the survival of bacilli under attack by the host immune system (Wayne and Sohaskey, 2001), such as macrophages (Kundu and Basu, 2021), tolerance to antibiotics (Hu et al., 2003; Nuermberger et al., 2004; Gengenbacher and Kaufmann, 2012) and recurrence (Gengenbacher and Kaufmann, 2012). Therefore, dormancy/resuscitation is the primary cause of the challenges associated with the clinical treatment of tuberculosis (Gupta et al., 2018), such as prolonged treatment cycles (Zhang, 2014), poor treatment efficacy (Wayne and Hayes, 1996), and difficulties in disease control (Gupta et al., 2018).

Among the approximately 400 dormancy/resuscitation genes identified thus far (Kundu and Basu, 2021), genes involved in DNA replication (Nandi et al., 2019); translation (Nandi et al., 2019); fatty acid and mycolic acid biosynthesis (Salina et al., 2014); primary metabolism (Betts et al., 2002); and cell wall biosynthesis (Iona et al., 2016) were downregulated, while genes involved in fatty acid degradation (Muñoz-Elías and McKinney, 2005; Del Portillo et al., 2018); the transporter systems of molybdate, phosphate and other nutrients (Betts et al., 2002; Muñoz-Elías and McKinney, 2006); iron storage proteins (Voskuil et al., 2004); mycobactin synthesis (Bacon et al., 2004); and toxin/antitoxin systems (Aguilar-Ayala et al., 2017; Hudock et al., 2017) were upregulated in dormant bacilli. Correspondingly, during resuscitation, genes involved in aerobic respiration (Salina et al., 2014), DNA replication (Nandi et al., 2019), expression regulation (Rodríguez et al., 2014; Nandi et al., 2019), cell division (Iona et al., 2016), mycolic acid and lipoarabinomannan (LAM) synthesis, phthiocerol mycocerosate (PDIM) and sulfolipid (Kundu and Basu, 2021), and ribosome biosynthesis were upregulated, in addition to five essential resuscitation promoting factors (Rpfs) (Iona et al., 2016). Then, the structure of the peptidoglycan (PG) layer was reconstituted by PG lytic transglycosylases to meet the metabolic requirements of cell growth and division (Downing et al., 2005; Tufariello et al., 2006; Kondratieva et al., 2011; Gengenbacher and Kaufmann, 2012). Therefore, neither dormancy nor resuscitation can occur on wheels without global transcriptional regulator(s).

DosR-DosS (or DevR-DevS), which is thought to be a potential regulator of dormancy/resuscitation (Leistikow et al., 2010; Schubert et al., 2015) because it responds to hypoxia (Sherman et al., 2001), NO (Voskuil et al., 2003) and CO (Kumar et al., 2008). However, DosR is dispensable for *M. tuberculosis*, and no more than 50 target genes have been identified thus far (Park et al., 2003); moreover, the survival rate of the DosR knockout mutant was only slightly lower than that of the wild type under hypoxic conditions (Rustad et al., 2008, 2009; Taneja et al., 2010).

Currently, dormancy and persistence, two highly similar reversible states of low metabolism and growth arrest, are mainly distinguished by growth-related features (Kell et al., 2015); for example, dormant tubercle bacilli must undergo resuscitation under specific conditions before being cultured *in vitro* (Kell et al., 1998), whereas persistent cells can be cultured *in vitro* immediately (Joshi et al., 2021). Research on their discrepancies and associations could undoubtedly make significant contributions to the understanding of *M. tuberculosis* (Zhang, 2004; Lipworth et al., 2016).

MtrA, the response regulator of MtrA/B, which is the only essential two-component system of *M. tuberculosis* (Zahrt and Deretic, 2000), was revealed to be one of five essential genes of *M. tuberculosis* under all *in vivo* (Sassetti and Rubin, 2003; Rengarajan et al., 2005) and *in vitro* (Griffin et al., 2011; DeJesus et al., 2017; Minato et al., 2019) conditions by the transposon mutagenesis strategy. It is highly conserved in the *Mycobacterium* genus, even for *M. leprae*, which has lost most of its sigma factors and two-component systems (Tyagi and Sharma, 2004). MtrA has been shown to be associated with cell division, cell wall synthesis, antibiotic resistance, cell morphology and osmoprotection not only in *M. tuberculosis* (Gorla et al., 2018; Peterson et al., 2023) but also in *M. smegmatis* (Gorla et al., 2018), *M. avium* (Cangelosi et al., 2006), *M. leprae* (Marques et al., 1998) and even other actinobacterial species (Brocker et al., 2011).

MtrA not only binds to *oriC* in *M. tuberculosis* (Rajagopalan et al., 2010) but also regulates essential resuscitation-promoting factors (*rpfA*, *rpfB*, and *rpfC*) (Sharma et al., 2015; Chatterjee et al., 2018), *dnaA* (DNA replication) (Li et al., 2010), and *wag31* (cell division) (Gorla et al., 2018). Furthermore, the intracellular survival rate of *M. tuberculosis* inside THP-1 cells was significantly reduced after MtrA was overexpressed (Fol et al., 2006), and slightly knocking down the expression of MtrA could considerably sensitize *M. tuberculosis* to vancomycin, bedaquiline, isoniazid and rifampicin (Peterson et al., 2023) and inhibit its cell division and growth (Peterson et al., 2021, 2023). Additionally, *whiB4*, an oxidative stress regulator (Chatterjee et al., 2018), and *fbpB* (Ag85B), which are responsible for cell adhesion (Rajagopalan et al., 2010), are regulatory targets of MtrA (Chatterjee et al., 2018). The above reports strongly suggested an association between MtrA and dormancy/resuscitation.

To date, the number of *M. tuberculosis* MtrA target genes reported ranged from 45 (Chatterjee et al., 2018) to 279 (Minch et al., 2015; Gorla et al., 2018), and only 15 genes were supported by all three studies utilizing chromatin immunoprecipitation sequencing (ChIP-seq), the mainstream research technique currently exploring the regulons of transcription factors, due to its high false positive and negative rates and unsatisfactory reproducibility (Park, 2009; Lihu and Holban, 2015). This study explored the roles and functions of *M. tuberculosis* MtrA by revealing its regulons according to verified genome-wide binding sites.

## 2 Materials and methods

### 2.1 Reagents

The majority of reagents were purchased from Sigma-Aldrich (St. Louis, MO, USA).

### 2.2 Bacterial strains, culture and transformation

The bacterial strains used in this study, *M. tuberculosis* H37Rv, *M. smegmatis* MC2-155 and *Escherichia coli* Top10 and BL21 (DE3), were stocked in our laboratory.

*E. coli* was incubated with LB media at 37°C and 220 RPM. *M. tuberculosis* H37Rv and *M. smegmatis* MC2-155 were cultured in Middlebrook 7H9 broth supplemented with 10% OADC and 0.05% Tween 80 at 37°C and 200 RPM or cultured on 7H10 supplemented with 50 μg/ml kanamycin when needed.

The preparation and transformation of electrocompetent *M. smegmatis* cells were performed as previously reported (Jacobs et al., 1991).

### 2.3 Construction of plasmids

The *eGFP* fragment from the plasmid pUC-eGFP was PCR amplified using the primers EGFP-F (5′- gctaggatccATGGTGAGCAAGGGCGAGG -3′) and EGFP-R (5′- ctacgtcgacTTACTTGTACAGCTCGTCCATGCC -3′), which were subsequently ligated with pMV261 to construct pMV261-eGFP after both fragments were double digested by *Bam*HI and *Sal*I.

**FIGURE 1:**
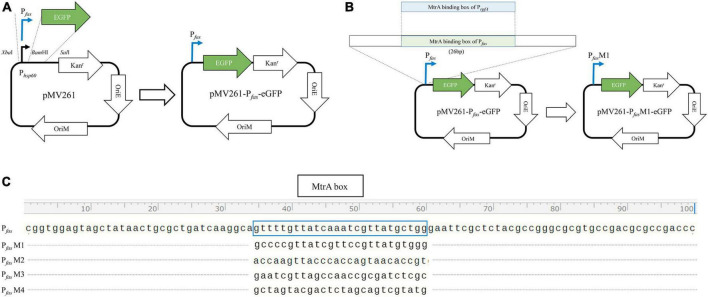
Diagram of the construction of the reporter plasmid. **(A)** Construction diagram of pMV261-pfas-eGFP. **(B)** Diagram of MtrA binding box replacement. **(C)** The MtrA binding site (boxed) of *fas* (KD = 4.62E-08 M) was replaced with those of *rpfA* (KD = 1.35E-07 M, P*_*fas*_*M1), *fadE5* (KD = 1.42E-05 M, P*_*fas*_*M2), *fbpC* (KD = 9.85E-03 M, P*_*fas*_*M3) and *carA* (KD > 0.01 M, P*_*fas*_*M4).

PCR-amplified promoters of MtrA target genes, such as *fas*, were ligated with *Xba*I and *Bam*HI double-digested pMV261-eGFP to replace the hsp60 promoter (398 bp) to yield pMV261-P*_*fas*_*-eGFP (Figure 1A). All of the primers used are listed in Table 1.

**TABLE 1:** Primers for amplifying the promoter region of MtrA target genes.

Promoter	Direction	Sequence (5′ → 3′)
P*_*fas*_*	Forward	gtactctagaCCTGCCGCCCCGAGCCTG
	Reverse	gcatggatccGGTCTATGTCTCCCTATGT GCATCG
P*_*rpfA*_*	Forward	gtactctagaGGCCATGTGACATT ACCCACA
	Reverse	gcatggatccACGTTAGGTAATTCCTCT CGGTACA
P*_*rpfB*_*	Forward	gtactctagaTGAGCGCGC CGAGCGGCC
	Reverse	gcatggatccCAGCAGCGCACCGACTACC
P*_*katG*_*	Forward	gtactctagaGCCGCGGGC TTGGTGCGA
	Reverse	gcatggatccCCCACGACGGGACAGCCG
P*_*ripA*_*	Forward	gtactctagaTTGATGCAACTGCGCGGG
	Reverse	gcatggatccGACAAACCGTGCGGCCGG
P*_*mcdR*_*	Forward	gtactctagaGTCACCTGACGATTTCAAGG
	Reverse	gcatggatccTGGCTCCCCCGACAGCCA
P*_*fbpB*_*	Forward	gtactctagaATTCTCGCACCTCAATCGTTCC
	Reverse	gcatggatccACTTGTGCCCCTTTGTCCTGT
P*_*ppgs*_*	Forward	gtactctagaCGGCGTCGTAGGTCCATG
	Reverse	gcatggatccTTAAGCCCACAAGTTACACGTTG
P*_*rpfC*_*	Forward	gtactctagaCTGTCGCAGATTTGGAGTCACTG
	Reverse	gcatggatccAGAGCGACATGGATGCCGT
P*_*fbpC*_*	Forward	gtactctagaGTCACCCAGTCGCTCTGCC
	Reverse	gcatggatccAGCTACTACCAATCCCAACT CTCAT

The MtrA-binding fragment (26 bp) from *rpfA*, *fadE5*, *fbpC*, and *carA(*no binding*)* with a 15 nt single-stranded homologous arm on each side was recombined with PCR-amplified pMV261-P*_*fas*_*-eGFP (pRP*_*fas*_*-F:5′- tgccttgatcagcgcagttatagc -3′, and pRP*_*fas*_*–R: 5′- gaattcgctctacgccgggc -3′) using Exnase II (Vazyme, Nanjing, China) to replace the binding fragment of P*_*fas*_* (Figures 1B, C). All constructs were used for subsequent experiments after sequencing verification.

### 2.4 Verification of promoter activity in vivo

Bacterial cells of *M. smegmatis* confirmed transformants growing to OD600 = 0.6 were harvested by centrifugation (5000 × g, 10 min at 4°C), washed and resuspended in PBS. Then, the concentration of the cells (diluted 10-fold) was adjusted to OD600 = 0.7. The eGFP expression of cells was assayed by measuring the fluorescence using a multifunction microplate reader (Tecan Infinite F200, λ_*ex*_: 488 nm, λ_*em*_: 507 nm) in a flat bottom 96-well black plate (Falcon).

### 2.5 MtrA expression, purification and binding affinity assay

MtrA expression, purification and binding affinity assays by biolayer interferometry (BLI) were conducted following previous reports (Memon et al., 2023).

### 2.6 Genome-wide mining for MtrA binding sites

Fifty-two potential ca. 200-bp binding fragments of MtrA retrieved according to the ChIP-seq data (Minch et al., 2015; Chatterjee et al., 2018; Gorla et al., 2018) were amplified using *M. tuberculosis* H37Rv genomic DNA as a template, primers were designed using Primer3 (Untergasser et al., 2012), and the sequences are listed in Supplementary Table 1.

The sequences verified by EMSA (Supplementary Figure 1) were further analyzed by MEME (Bailey and Elkan, 1994) to identify conserved motifs. Then, the minimum length of the binding fragment was determined utilizing BLI to assay the affinity of the sequentially truncated fragments (Supplementary Table 2). The position weight matrix (PWM) was established by MEME (Bailey and Elkan, 1994) by analyzing twenty-six high-affinity (KD < 5.77E-06 M) 26-bp fragments. Then the genome of *M. tuberculosis* H37Rv (AL123456) was comprehensively searched for potential binding sites (*q*-value ≤ 0.5) by FIMO (Grant et al., 2011) utilizing the established PWM. All potential binding sites identified were further verified by BLI, and all fragments were synthesized by Tsingke Biotechnology (Shanghai, China). The consensus sequence of MtrA binding fragments was generated by WebLogo (Crooks et al., 2004).

### 2.7 Gene annotation and gene enrichment analysis

The genes closest to binding sites were annotated by Tuberculist (Lew et al., 2011), TBDB (Galagan et al., 2010) and BioCyc (Karp et al., 2019). The protein families were categorized by COG (Galperin et al., 2021), and the orthology clusters were determined by BlastKOALA (Kanehisa et al., 2016). Gene enrichment (the proportion of MtrA target genes within specific pathway) was analyzed using KOBAS-i (Bu et al., 2021); the node size represents the *P* value, and the edge represents a correlation value greater than 0.35.

## 3 Results

### 3.1 Promoters were functional in vivo, and 288 target ORFs were predicted according to 294 binding sites identified

The conserved motif of MtrA binding fragments were identified using MEME by analyzing the sequences of 52 binding fragments verified by EMSA (Supplementary Figure 1) from 76 reported ones supported by three independent ChIP-seq experiments (Minch et al., 2015; Chatterjee et al., 2018; Gorla et al., 2018). After the minimum length of binding fragment of MtrA was determined to be 26 bp through assaying affinity (BLI) of MtrA binding with the sequentially truncated fragments. 294 binding sites were confirmed to be bound by MtrA (KD < 1.00E-3 M) (Supplementary Table 1) from 355 potential binding sites (Supplementary Data) predicted by FIMO utilizing the position weight matrix (PWM) established by MEME using 26 sequences of high-affinity (KD < 5.77E-06 M) 26-bp fragments from the whole genome of *M. tuberculosis* H37Rv.The conserved MtrA motif generated from sequences of 294 binding sites consisting of two 7-bp degenerate direct repeats of t/c-G/t-T/a-n-a-C/T-c (MtrA binding box) with 4-bp spacer (Supplementary Figure 2), was significant similar with the results based on DNaseI footprinting of *oriC* and P*fbpB* (Rajagopalan et al., 2010) and ChIP-seq analysis (Minch et al., 2015; Gorla et al., 2018) with some differences in the abundance of bases at specific locations, for example, the proportion of 7*^th^* T and 9*^th^* A in the fragment was significantly higher than that of reports (Rajagopalan et al., 2010; Gorla et al., 2018).

**FIGURE 2:**
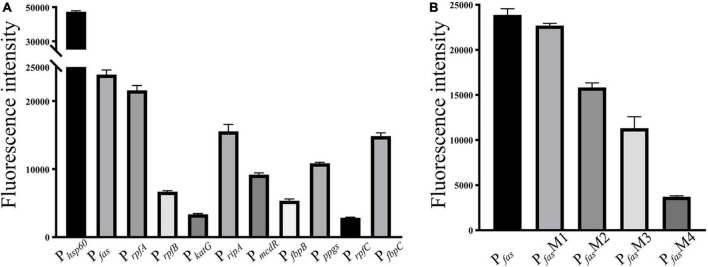
Promoter *in vivo* activity assay. **(A)** Selected promoters of MtrA target genes. **(B)** Effect of replacement of the MtrA binding box in the *fas* promoter.

As shown in Figure 2, 10 randomly selected promoters of MtrA target genes could drive the expression of the reporter gene individually (Figure 2A); furthermore, the sequence of the 26-bp fragment of the MtrA binding site significantly influenced the expression of the reporter gene (Figure 2B). According to 294 binding sites confirmed, 288 ORFs were assigned as potential MtrA target genes, accounting for 7.2% of the total number of *M. tuberculosis* H37Rv ORFs. A total of 39.1% of the binding sites were found in noncoding regions, including *oriC*, and six ORFs had two binding sites identified to be closest to them (Table 2, Supplementary Data).

**TABLE 2:** Distribution of MtrA binding sites and annotation of the predicted ORF.

Binding sites			ORFs		
**No.**	**Distribution**		**No.**	**Annotation**	
	**Non coding**	**Coding**		**Anno tated**	**Unknown**
294	115	179	288	270	18

**FIGURE 3:**
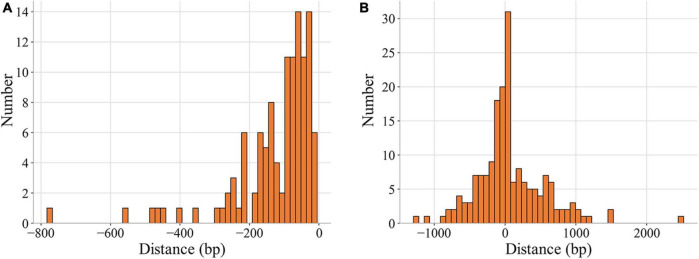
Distribution of the distances of MtrA binding sites to target genes. The distance represents the number of base pairs between the first nucleotide of the binding site and the start codon (0 on the X axis) of the target gene **(A)** noncoding region. **(B)** Coding region.

As shown in Figure 3, the percentages of binding sites in noncoding and coding regions whose distances to their target genes were shorter than 200 bp were 83.9% and 48.6%, respectively. A total of 65.6% of the binding sites (193) were located in regions upstream of target genes, and the rest were in the target genes (Table 2, Supplementary Data). Among the 193 binding sites located in regions upstream of target genes, 57.0% were in intergenic regions, and 43.0% were in genic regions of the preceding gene.

A total of 127 distinct KEGG ortholog groups (KOs) were identified in 270 annotated ORFs (93.8%), and most KO categories contained only 1 or 2 genes, except for K06994, which comprised 6 ORFs coding for RND superfamily drug exporters. The functions of MtrA target genes are highly diverse and include various enzymes, PE/PPE family proteins, toxin-antitoxin (TA) systems, transport/secretion systems, transcriptional regulation factors, protein kinases, etc., and even MtrA itself (Supplementary Table 3).

### 3.2 Genes related to cell wall biogenesis, lipid metabolism, cell division, DNA replication and damage repair were the main targets of MtrA

**FIGURE 4:**
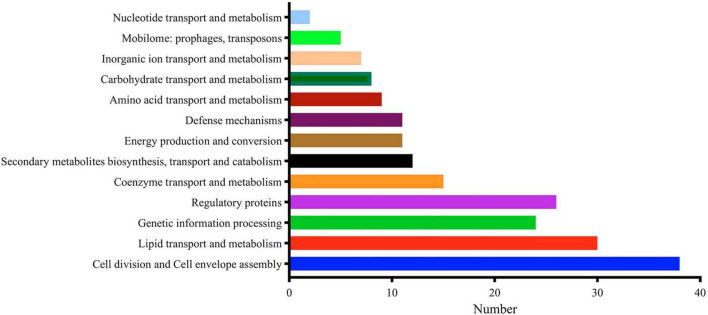
Major functional categories of MtrA targets.

MtrA target genes involved in the initiation and elongation of chromosome replication (*dnaA*, *dnaB*, *holB*, *dnaN*), four of nine *M. tuberculosis* DNA repair systems, such as translesion synthesis and base excision repair (*dnaE2*, *nth*, *mpg* and *ligD*); cell wall synthesis, modification and maintenance of cell morphology (*murJ*, *ldtA*, *ponA2*, *rpfA-E*, *aftC*, *fas*, *ppgS*, *fbpA-C, etc.*); LAMs, PDIMs and other important cell wall lipid synthesis (*pimE*, *mptC*, *capA*, *fadD28-29*, *ppsE*, *etc.*); and divisome formation, cell separation, cell division regulation and other cell division processes (*sepF*, *ftsW*, *wag31*, *cwlM*, *ami1*, *ripA*) accounted for 17.01% of the total number of regulons (Supplementary Table 3). As shown in Figure 4, regulatory factors (26) were also the primary targets of MtrA, including 8 transcription factors from the WhiB (whiB1, whiB3, whiB4 and whiB7) and sigma factor families (sigB, sigD, sigE and sigH), three members of the Ser/Thr protein kinase family (pknA, pknF, and pknH) (Supplementary Table 3), cell division and cell envelope assembly (38), lipid transport and metabolism (30) and genetic information processing (24).

### 3.3 32.6% of MtrA target genes were essential genes

Among the MtrA regulons, 94 (32.6%) genes were essential genes reported (Sassetti and Rubin, 2003; Rengarajan et al., 2005; Griffin et al., 2011; DeJesus et al., 2017; Minato et al., 2019), including 25 *in vivo* and 74 *in vitro* essential genes (Table 3). Interestingly, among the MtrA target genesthat were essential under any one of five *in vivo* or *in vitro* experimental conditions, only 4 genes were indispensable in both murine spleens (Sassetti and Rubin, 2003) and macrophages (Rengarajan et al., 2005), accounting for 28.6% and 26.7%, respectively, of the total number of essential genes. However, 40 genes were essential for all three *in vitro* experimental conditions, accounting for 76.9%, 81.6% and 60.6%, respectively, of the total number of essential genes (Supplementary Table 3). however, only MtrA was indispensable under all *in vivo* and *in vitro* experimental conditions (Supplementary Table 3).

**TABLE 3:** Essential genes regulated by MtrA.

	*In vivo* (mouse)		*In vitro*		
	**Spleen (Sassetti and Rubin, 2003)**	**Macrophages (Rengarajan et al., 2005)**	**Minato (Minato et al., 2019)**	**DeJesus (DeJesus et al., 2017)**	**Griffin (Griffin et al., 2011)**
No.	14	15	52	49	66

### 3.4 The primary carbohydrate, lipid, nucleotide and amino acid metabolic pathways were the main regulatory targets of MtrA

**FIGURE 5:**
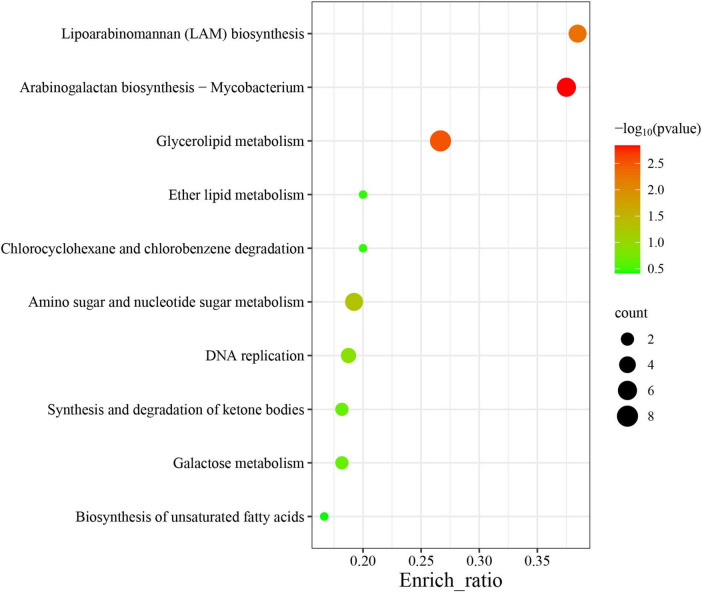
Top 10 enriched pathways of MtrA target genes. The abscissa label represents the enrichment ratio of pathways, which is calculated as the input gene number/background gene number.

Among the 60 pathways in which MtrA target genes were found, primary metabolism pathways were predominant (83.3%), including 11 carbohydrate metabolism pathways, 7 lipid metabolism pathways, 2 nucleotide metabolism pathways, 11 amino acid metabolism pathways, and 3 glycan biosynthesis and metabolism pathways, followed by genetic information processing pathways (11.7%), including 4 replication and repair pathways, 2 translation pathways and 1 folding, sorting and degradation pathway. The remaining pathways were mainly associated with environmental information processing and cellular process pathways (Table 4). Furthermore, the gene enrichment ratios of the LAM biosynthesis (38.5%) and arabinogalactan biosynthesis (37.5%) pathways were approximately two times greater than those of the other pathways (Figure 5).

**TABLE 4:** Comparison among MtrA target pathways, dormancy/resuscitation pathways and persistence pathways.

Functional category	Pathway	Function	Dormancy/ Resuscitation (Schubert et al., 2015; Iona et al., 2016; Mittal et al., 2020; Kundu and Basu, 2021)	MtrA	Persistence (Wang et al., 2011; Joshi et al., 2021)
Carbohydrate metabolism	mtu00640	Propanoate metabolism	+	+	+
	mtu00520	Amino sugar and nucleotide sugar metabolism	+	+	+
	mtu00562	Inositol phosphate metabolism	+	+	−
	mtu00650	Butanoate metabolism	+	+	+
	mtu00630	Glyoxylate and dicarboxylate metabolism	+	+	+
	mtu00660	C5-Branched dibasic acid metabolism	+	+	−
	mtu00620	Pyruvate metabolism	+	+	+
	mtu00051	Fructose and mannose metabolism	+	+	+
	mtu00052	Galactose metabolism	+	+	+
	mtu00010	Glycolysis / Gluconeogenesis	+	+	+
	mtu00020	Citrate cycle (TCA cycle)	+	+	+
	mtu00500	Starch and sucrose metabolism	+	−	+
	mtu00030	Pentose phosphate pathway	+	−	+
Energy metabolism	mtu00910	Nitrogen metabolism	+	+	+
	mtu00920	Sulfur metabolism	+	+	+
	mtu00680	Methane metabolism	+	−	+
	mtu00190	Oxidative phosphorylation	+	−	+
Lipid metabolism	mtu00561	Glycerolipid metabolism	+	+	+
	mtu00071	Fatty acid degradation	+	+	+
	mtu00061	Fatty acid biosynthesis	+	+	+
	mtu00072	Synthesis and degradation of ketone bodies	+	+	+
	mtu01040	Biosynthesis of unsaturated fatty acids	+	+	−
	mtu00564	Glycerophospholipid metabolism	−	+	−
	mtu00565	Ether lipid metabolism	−	+	−
Nucleotide metabolism	mtu00230	Purine metabolism	+	+	+
	mtu00240	Pyrimidine metabolism	+	+	−
Amino acid metabolism	mtu00380	Tryptophan metabolism	+	+	+
	mtu00310	Lysine degradation	+	+	+
	mtu00270	Cysteine and methionine metabolism	+	+	+
	mtu00280	Valine, leucine and isoleucine degradation	+	+	+
	mtu00250	Alanine, aspartate and glutamate metabolism	+	+	+
	mtu00360	Phenylalanine metabolism	+	+	+
	mtu00340	Histidine metabolism	−	+	+
	mtu00220	Arginine biosynthesis	+	−	+
	mtu00260	Glycine, serine and threonine metabolism	+	−	+
	mtu00350	Tyrosine metabolism	−	+	−
	mtu00290	Valine, leucine and isoleucine biosynthesis	−	+	−
	mtu00300	Lysine biosynthesis	−	+	−
Metabolism of other amino acids	mtu00410	beta-Alanine metabolism	+	+	+
	mtu00450	Selenocompound metabolism	+	−	+
	mtu00430	Taurine and hypotaurine metabolism	+	−	+
	mtu00480	Glutathione metabolism	+	−	−
Glycan biosynthesis and metabolism	mtu00572	Arabinogalactan biosynthesis−Mycobacterium	+	+	+
	mtu00550	PG biosynthesis	−	+	+
	mtu00571	LAM biosynthesis	−	+	+
Metabolism of cofactors and vitamins	mtu00860	Porphyrin and chlorophyll metabolism	+	+	+
	mtu00790	Folate biosynthesis	+	+	+
	mtu00740	Riboflavin metabolism	+	+	−
	mtu00760	Nicotinate and nicotinamide metabolism	+	−	+
	mtu00780	Biotin metabolism	+	−	+
Metabolism of terpenoids and polyketides	mtu00903	Limonene and pinene degradation	+	+	+
	mtu01053	Biosynthesis of siderophore group nonribosomal peptides	+	+	+
	mtu00900	Terpenoid backbone biosynthesis	+	+	+
	mtu00281	Geraniol degradation	+	+	+
	mtu00523	Polyketide sugar unit biosynthesis	−	−	+
Biosynthesis of other secondary metabolites	mtu01130	Biosynthesis of antibiotics	+	+	+
	mtu00521	Streptomycin biosynthesis	+	−	+
	mtu00261	Monobactam biosynthesis	+	−	−
Xenobiotics biodegradation and metabolism	mtu00983	Drug metabolism−other enzymes	+	+	+
	mtu00930	Caprolactam degradation	+	+	+
	mtu00362	Benzoate degradation	+	+	+
	mtu00627	Aminobenzoate degradation	+	+	+
	mtu00984	Steroid degradation	−	−	+
	mtu00361	Chlorocyclohexane and chlorobenzene degradation	−	+	−
	mtu00625	Chloroalkane and chloroalkene degradation	−	+	−
Genetic Information Processing	mtu04122	Sulfur relay system	+	+	+
	mtu03010	Ribosome	+	+	+
	mtu03430	Mismatch repair	+	+	−
	mtu00970	Aminoacyl-tRNA biosynthesis	+	+	−
	mtu03030	DNA replication	+	+	−
	mtu03410	Base excision repair	+	+	−
	mtu03440	Homologous recombination	+	+	−
	mtu03018	RNA degradation	+	−	+
	mtu03420	Nucleotide excision repair	+	−	−
Environmental Information Processing	mtu02020	Cell division and cell wall two component system MtrA	−	+	+
	mtu02010	Phosphate ABC transporter	+	+	+
		Molybdate ABC transporter	+	+	−
Cellular Processes	mtu02024	Quorum sensing	+	+	+

**FIGURE 6:**
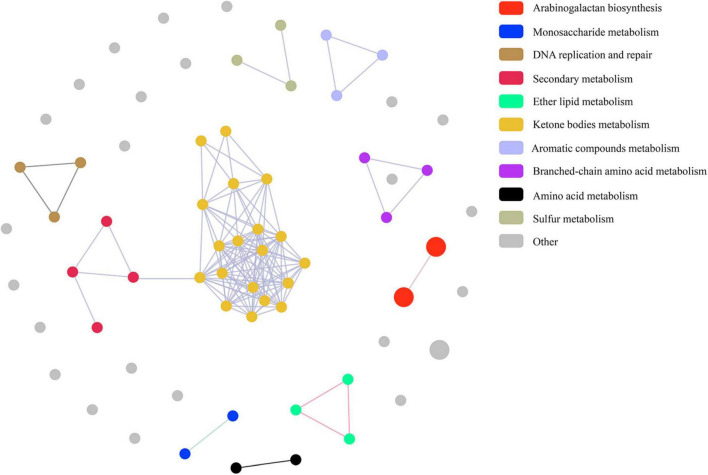
Pathway association among MtrA target pathways.

Pathway correlation analysis revealed that 37 MtrA target pathways comprised 10 distinct functional clusters, including arabinogalactan biosynthesis pathways (cluster 1), monosaccharide metabolism pathways (cluster 2), and DNA replication and repair pathways (cluster 3), and the remaining 23 pathways were orphan pathways (Figure 6). Although the majority of clusters were composed of 2-4 pathways, there were 19 pathways in cluster 6, including 5 carbon metabolism pathways and 4 amino acid metabolism pathways. Furthermore, the average number of genes per pathway in cluster 1 was 7, while that in the remaining clusters ranged from 1 to 4 (Figure 6). Interestingly, 59 MtrA regulons were reported to be relevant to dormancy/resuscitation, accounting for 13.9% of the total number of target genes of MtrA, including seven well-documented genes involved in dormancy/resuscitation, such as *rpfA*, *rpfB*, *rpfC* and *dnaA* (Supplementary Table 3).

### 3.5 MtrA target genes were identified in 75.4% pathways related to dormancy/resuscitation and persistence

Among 65 dormancy/resuscitation pathways predicted according to 425 dormancy/resuscitation genes reported thus far, such as carbohydrate metabolism, energy metabolism, lipid metabolism, replication and repair, etc., MtrA target genes were identified in 49 (75.4%) pathways, including 11 carbohydrate metabolism, 6 amino acid metabolism, 5 lipid metabolism, and 7 genetic information processing pathways (Table 4).

Among 57 persistence pathways predicted based on 159 reported persistence genes (Wang et al., 2011; Joshi et al., 2021), such as *ripA*, *fbpC* and other well-documented genes, MtrA target genes were identified in 43 pathways (75.4%), including 9 carbohydrate, 7 amino acid and 4 lipid metabolism pathways etc. (Table 4).

## 4 Discussion

Identifying target genes of prokaryotic global transcription factors that are crucial for revealing their functions remains a challenging task.

Among the 355 binding sites predicted in the genome of *M. tuberculosis* H37Rv using this study strategy, 82.8% (294) could be bound by MtrA with affinities (KDs) ranging from 10^–8^ to 10^–3^ M. According to the position of the binding sites in the genome, 288 target genes were predicted, including 17 out of the 18 genes regulated by MtrA reported thus far, excluding *rpoB* (Fol et al., 2006; Rajagopalan et al., 2010; Plocinska et al., 2012; Sharma et al., 2015; Chatterjee et al., 2018; Gorla et al., 2018).

For the MtrA target genes reported by three ChIP-seq studies (Minch et al., 2015; Chatterjee et al., 2018; Gorla et al., 2018), those supported by all three independent ChIP-seq studies were 100% consistent with our results; however, those supported by any two or one showed 65.6% and 32.9% overlap with our findings, respectively, which demonstrated the good performance of our strategy in identifying target genes of bacterial global transcription factors.

Since 7 bp motif used by Li et al. to mine for MtrA target genes was generated from one promoter (*dnaA*) through DNaseI footprinting and SPR analysis, only 14 of their 155 predicted MtrA target genes of *M. tuberculosis* were overlapped with our results, and for 264 potential MtrA target genes of *M. smegmatis*, the expression of less than 50% (12 out of 26 genes) of the genes assayed was significantly changed when MtrA was knocked down in *M. smegmatis* (Li et al., 2010). The aforementioned results further demonstrated that the attainment of successful genome-wide binding site identification for global transcription factors heavily relied on the acquisition of high-quality binding motif(s), which could be achieved only by analyzing a substantial number of promoters.

Since the coding region accounted for 90.7% of the genome of *M. tuberculosis* H37Rv (Lew et al., 2011), the probability of MtrA binding sites in the noncoding regions was 4.2-fold greater than the theoretical value, which indicated a strong bias in their distribution. Moreover, 32.7% of binding sites were found inside the target genes, suggesting complex regulatory mechanisms and functions since this kind of binding site can influence transcription, splicing and translation (Hua et al., 2022), and many target genes are associated with virulence, drug resistance, *etc.* (Galagan et al., 2013a,b; Stephanie et al., 2022).

As Supplementary Table 3 shows, the regulatory genes suggested that MtrA was involved in DNA replication (*dnaA*, *oriC*) (Ditse et al., 2017), DNA damage repair (*dnaE2, mku*, *ligD*) (Singh, 2017), cell division and cell wall assembly (*wag31*, *ripA* and *fbpB*) (Rajagopalan et al., 2010; Peterson et al., 2023) and even associated with virulence through stress adaptation, such as nutrient deprivation (Betts et al., 2002), oxidation (Narayan et al., 2007), acidity (Rodrigue et al., 2006), and antibiotics (Alam et al., 2009) (*whiB1*, *whiB3*, *whiB4*, *whiB7*, *sigB*, *sigD*, *sigE*, *sigH*, *pknA*, *pknF*, *pknH, etc.*).

A total of 32.6% (94) of the MtrA regulons were essential, and MtrA itself was essential under both *in vivo* and three *in vitro* experimental conditions (Sassetti and Rubin, 2003; Rengarajan et al., 2005; Griffin et al., 2011; DeJesus et al., 2017; Minato et al., 2019), which not only provided new explanations and insight into its indispensability but also demonstrated that the dynamic and precise regulation of MtrA was crucial in all stages of disease, from infection to survival and growth inside host cells.

The number of *in vivo* MtrA regulatory essential genes was significantly less than that *in vitro*, and the consistency of *in vivo* essential genes under different conditions was much lower than that *in vitro* (Table 3). These findings suggested that the indispensability of genes was significantly contingent upon the growing conditions and environment of tubercle bacilli and provided further evidence to support the hypothesis that *M. tuberculosis* evolved into a highly specific human pathogen (Gagneux, 2018; Pepperell, 2022).

The above findings not only indicated that the regulon study of global transcription factors through genome-wide binding profile investigations could comprehensively reveal their functions but also raised one question: why has *M. tuberculosis* MtrA evolved to regulate such large number of genes with diverse functions?

Several crucial genes involved in dormancy/resuscitation (Downing et al., 2005; Tufariello et al., 2006; Kondratieva et al., 2011) are regulated by MtrA, including resuscitation-promoting factors (*rpfA*, *rpfB*, and *rpfC*) (Sharma et al., 2015; Chatterjee et al., 2018), the replication initiation protein *dnaA* (Fol et al., 2006), the cell division protein *wag31* (Plocinska et al., 2012), the mycolyltransferase *fbpB* (Rajagopalan et al., 2010) and transcription factors such as *sigD* (Gorla et al., 2018) and *whiB3* (Gorla et al., 2018). After more dormancy/resuscitation-related genes were added to the regulatory list of MtrA in this study, MtrA regulatory genes were found to be involved in 75.4% of the dormancy/resuscitation pathways predicted according to the genes reported thus far (Schubert et al., 2015; Iona et al., 2016; Mittal et al., 2020; Kundu and Basu, 2021), from carbohydrate metabolism, energy metabolism, lipid metabolism, DNA replication and repair pathways to virulence pathways, such as *ldtA*, *ldtC* (Tolufashe et al., 2020), *ppgS*, *rv3779* (Draper et al., 1997; Bhamidi et al., 2008; Skovierová et al., 2010; Wheat et al., 2015), *capA*, and *mptC* (Maeda et al., 2003; Kang et al., 2005), which respond to the long-term survival of tubercle bacilli inside macrophages by inhibiting phagolysosome maturation and infecting dendritic cells (Table 4, Supplementary Table 3).

Modulating dormancy/resuscitation is the most reasonable and plausible explanation for why MtrA regulating approximately 300 genes involved in DNA replication, DNA damage repair, cell division, stress tolerance to the primary metabolism of carbohydrates, lipids, amino acids, etc.

Furthermore, among the 53 mutual pathways between dormancy/resuscitation and persistence, which accounted for 81.5% and 93.0% of the total number of pathways, respectively, energy metabolism, lipid metabolism, amino acid metabolism, environmental information processing, and MtrA target genes were identified in 73.6% of the pathways (39) (Table 4).

As Table 4 shows, the cellular processes and representative pathways of latent tuberculosis, such as fatty acid degradation (25), glyoxylate shunting (102) and gluconeogenesis (103), were exactly identical between dormancy/resuscitation and persistence, and they were all regulated by MtrA. Approximately 80.0% of the pathways related to xenobiotic biodegradation and metabolism, terpenoid and polyketide metabolism, and lipid and carbohydrate metabolism were shared between dormancy/resuscitation and persistence, and more than 80.0% of these pathways were regulated by MtrA. The fact that dormancy/resuscitation shares the majority of its pathways mentioned above with persistence provides an explanation for why they are significantly similar in their cellular processes and physiological states (Kell et al., 2015).

However, for pathways related to genetic information processing, glycan biosynthesis and metabolism, only approximately 30% of the pathways were identical between dormancy/resuscitation and persistence (Table 4). Interestingly, the pathways crucial for resuscitation, such as DNA replication, homologous recombination repair, mismatch repair, nucleotide excision repair and RNA degradation (Gengenbacher and Kaufmann, 2012), were identified only in dormancy/resuscitation, and all of these pathways are regulated by MtrA. These results indicated that dormancy/resuscitation and persistence are unique pathways; for example, *rpfA*-*E* are essential for reconstituting the structure of the PG layer to meet the metabolic requirements of cell growth and division (Downing et al., 2005; Tufariello et al., 2006; Kondratieva et al., 2011; Gengenbacher and Kaufmann, 2012), and several genes involved in DNA repair pathways are crucial for repairing DNA damage accumulated during long periods of deep growth arrest (Salina and Makarov, 2022) when tubercle bacilli reinitiate growth during the process of resuscitation.

Additionally, MtrA plays an important role in tuberculosis (mtu05152), β-lactam resistance (mtu01501) and vancomycin resistance (mtu01502) since its binding sites were identified in the promoter region of several crucial genes in the PG biosynthesis pathway (*murA*, *murJ*, *ldtA*, *ldtC*, *ponA2*, *PBP-lipo* and *dacB1*, *etc*.) and LAM biosynthesis pathway (*pimE*, *mptB mptC*, and *capA*) (Supplementary Table 3).

For similar studies on *M. tuberculosis* MtrA employing ChIP-seq technique, 216 and 155 target genes were identified from exponential and stationary phases of *M. tuberculosis* by expressing phosphorylation competent MtrA_Y102C_ mutant protein (function independent of MtrB)(Gorla et al., 2018), and 93 mutual genes were shared by two phases, while only 89 (Minch et al., 2015) and 45 (Chatterjee et al., 2018) target genes were reported when wildtype MtrA was expressed. Since less than 1% purified MtrA expressed by *E. coli* was phosphorylated at positions like D13, D56 (Fol et al., 2006; Rajagopalan et al., 2010; Al Zayer et al., 2011), our previous work demonstrated that unphosphorylated MtrA could bind with its target DNA fragments after its dimerization was facilitated by the substrate DNA regardless of affinity (Memon et al., 2023). However, the dimerization of other members from OmpR subfamily, such as KdpE (Narayanan et al., 2014), PhoP (He et al., 2016) and PmrA (Lou et al., 2015), was facilitated by phosphorylation on regulatory domain (Gao and Stock, 2010; Barbieri et al., 2013). Interestingly, we recently found that phosphorylation could significantly increase (Rv1796, Rv1908c) or reduce (Rv2524c) the affinity of MtrA to its target fragment (data not show). The aforementioned results suggests that the affinity alterations of phosphorylated and unphosphorylated MtrA to its target fragments in fragment-specific manner are likely crucial for *M. tuberculosis* to adapt to diverse environmental conditions and stresses through dynamically adjusting the profile of MtrA target genes.

In summary, we should pay utmost attention to MtrA, as its potentially indispensable roles in tuberculosis and antibiotic resistance, especially in dormancy/resuscitation and persistence, indicate that it may be the Achilles heel of *M. tuberculosis*.

## Data availability statement

The original contributions presented in the study are included in the article/Supplementary material, further inquiries can be directed to the corresponding author/s.

## Author contributions

XF: Writing−review and editing, Writing−original draft, Investigation, Formal analysis. XW: Writing−review and editing, Supervision. AM: Investigation, Writing−review and editing. X-YF: Investigation, Writing−review and editing, Resources. QS: Writing−review and editing, Formal analysis. HC: Supervision, Writing−review and editing, Formal analysis. YY: Writing−review and editing, Supervision. ZD: Supervision, Conceptualization, Writing−review and editing. JM: Investigation, Writing−review and editing, Supervision. WM: Writing−original draft, Conceptualization, Writing−review and editing, Supervision, Resources, Funding acquisition.

## Supplementary material

Supplementary Table 1 Sequences of primers used for EMSA.

Supplementary Table 2 Effect of the fragment length of Rv0867c on MtrA binding affinity.

Supplementary Table 3 Summary of MtrA regulons.

Supplementary Figure 1 EMSA analysis of MtrA binding to selected promoters of target genes.

Supplementary Figure 2 MtrA logo diagram of the motif.

Supplementary Data MtrA binding sites.
