# Supplementary material for: Regulatory role of Mycobacterium tuberculosis MtrA on dormancy/resuscitation revealed by a novel target gene-mining strategy
Source: Front Microbiol. 2024 Jun 17;15:1415554. doi: 10.3389/fmicb.2024.1415554 (PMC11215152; doi:10.3389/fmicb.2024.1415554)
Supplement: Supplementary Table 1 — Sequences of primers used for EMSA. [file Presentation_1.pdf]

Supplementary Material

1 Supplementary Methods

Electrophoretic mobility shift assay (EMSA): EMSAs were generally performed as previously described (1) with some modifications. After MtrA (3 μM) binding with fragment (100 μM) for 30 min at 25 ℃, samples were separated by 6% native PAGE at 120 V for 60 min at 4 ℃, then gel was recorded using Molecular Imager (Bio-Rad, USA) after dyeing with 0.01% GelRed (Biotium, USA).

2 Supplementary Figures and Tables

2.1 Supplementary Figures

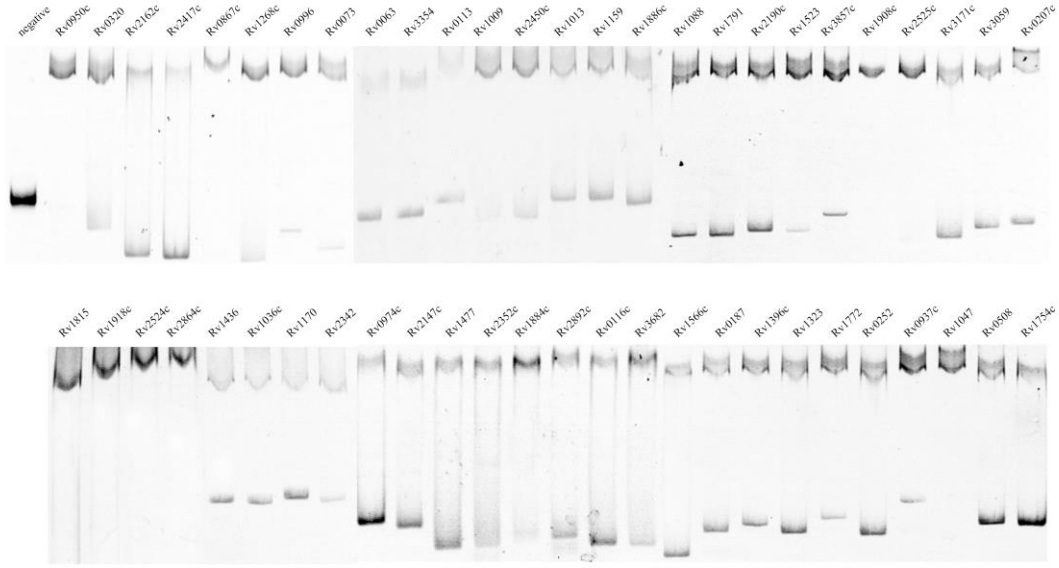

Supplementary Figure 1. EMSA analysis of MtrA binding to selected promoters of target genes.

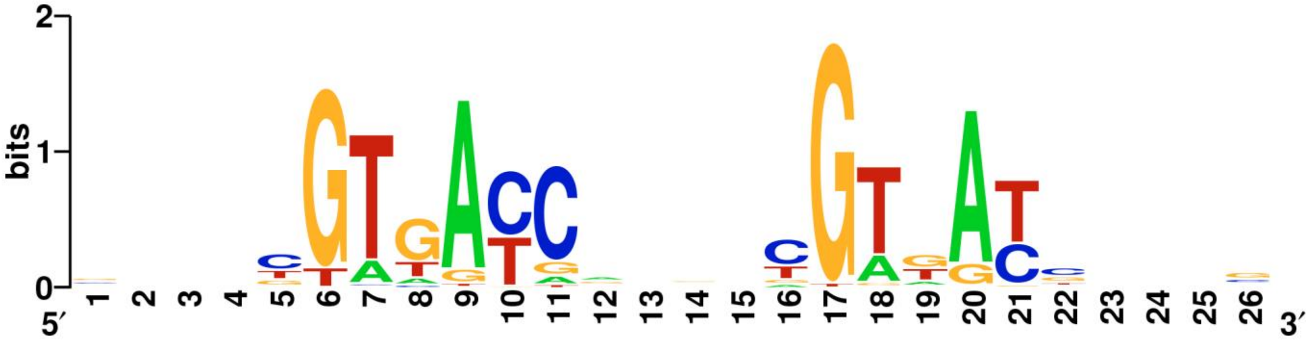

Supplementary Figure 2. MtrA logo diagram of the motif.

2.2 Supplementary Tables

Supplementary Table 1. Sequences of primers used for EMSA

Target	Direction	Sequence (5′→3′)
Negative	Forward	ggtggcgtccactcatacgt
	Reverse	ggattccgaccgcggatgga
Rv0950c	Forward	gatgcggccaacgatcgcga
	Reverse	gtccagccgaaacgtgatcc
Rv0320	Forward	gcagatatccgcgaaccgat
	Reverse	gtactgacatctccgcccgt
Rv2162c	Forward	gcgccgcaatcacaaacgac
	Reverse	gcctcctttggtcttgcagg
Rv2417c	Forward	gatttctcgttcggcacccc
	Reverse	gatcatcgcatcgctgccct
Rv0867c	Forward	gtcggcgtttccgtcacaat
	Reverse	gagatgacgccgtcgcgtta
Rv1268c	Forward	agcgcgaaggtggcggtctt
	Reverse	ttaggcagccgaccgatcgt
Rv0996	Forward	ggcgagaccatctgttgatg
	Reverse	ttcggttccgatccccgcga
Rv0073	Forward	gacgaagtcgacatcacgac
	Reverse	tcagatcaccgggtcgatga
Rv0063	Forward	gttcgttagccagtacgcca
	Reverse	ggcagcaccccgaattttgg
Rv3354	Forward	gcgaagaaacgtctggcgag
	Reverse	aaggccgcggcgctgagaat
Rv0113	Forward	gtgacgatatcgtgccggaa
	Reverse	gcaggacaaagacattcggt
Rv1009	Forward	gaagaggtggcgctcatcac
	Reverse	tatccccgacctcggcaaca
Rv2450c	Forward	cacgcgggtccgtagattca
	Reverse	tccgtgtccggggtcacaat
Rv1013	Forward	gccagctcacggcgatagct
	Reverse	gttgggtctcggttcggtga
Rv1159	Forward	gcggacaaacgtccagatgg
	Reverse	tttagcgatcgccgagcgca
Rv1886c	Forward	gaatctttcggctcacgtct
	Reverse	gcgattctgagaaatccgcg
Rv1088	Forward	ggttttcgatgcattggctg
	Reverse	gctggtgtggcaatcatgta
Rv1791	Forward	gccgataaatacgctggtca
	Reverse	gctctctgcaagagagtaga
Rv2190c	Forward	gattacacgcgcgatcaacc

	Reverse	gatgcccctggagccctata
Rv1523	Forward	atgacgatcagcggccatcg
	Reverse	gccagccgatcccacgaaat
Rv3857c	Forward	gtatcaaatccgagggcaca
	Reverse	gaacgagcgcccgataacca
Rv1908c	Forward	tcatatgacccacgacggga
	Reverse	taacggcttcctgttggacg
Rv2525c	Forward	gccgcgaatttgagcacatc
	Reverse	gttgggagcgtaggcgatca
Rv3171c	Forward	gtgggtctccgagcactcat
	Reverse	gccctcgacgattactgaga
Rv3059	Forward	gtcgtcaacgacgcacgctt
	Reverse	agtcctcgccaccaggtgaa
Rv0207c	Forward	ggtctctttcagagcaaccg
	Reverse	gttcccagtggtgtgctgca
Rv1815	Forward	gcaacacgtctgcgggatcg
	Reverse	ttgaactcccatccgtcggg
Rv1918c	Forward	gcctggtgctgatgaaaggt
	Reverse	gcctacgtcggtggtttaca
Rv2524c	Forward	ggccggcgcgtatgtgttga
	Reverse	ttcgcccgctgcacggcatt
Rv2864c	Forward	gcagcaaacctgaggtggct
	Reverse	gccttgctcgattgagcggt
Rv1436	Forward	tcggctcaattgtcagcatc
	Reverse	tagatcagtgacacccgccg
Rv1036c	Forward	attgaggccatcttcccagt
	Reverse	ggcaacaacatggcacaaac
Rv1170	Forward	tgacgtggtctgatggtggg
	Reverse	ggtgtgcatgaacaaacagc
Rv2342	Forward	gatcaggtctatcaagcggc
	Reverse	gaggttccacttggtcaccc
Rv0974c	Forward	ttggccagttcggcgttgat
	Reverse	gaagtcgaacgccaataccg
Rv2147c	Forward	gtaatcctccatgggagcca
	Reverse	gaagtcgccgtcaaacatgg
Rv1477	Forward	ggacgatctcaatcaaggcg
	Reverse	tagggctcaactcggaggga
Rv2352c	Forward	tcatcgcaaccccaaaccag
	Reverse	gagccgtgtagctgcggatt
Rv1884c	Forward	ggtggagagcgacatggatg
	Reverse	gcaacacagtcgtattgcca
Rv2892c	Forward	gatcccggacctgcgtacat
	Reverse	gcacaggatggactggcttg
Rv0116c	Forward	gcaccattagccggcgacac
	Reverse	gcggggctatccagaaactg
Rv3682	Forward	gatgcgggccgtacaacatc
	Reverse	gctcaagcaacatcccgagg

Rv1566c	Forward	gtctgccattccagccggaa
	Reverse	tcatcaccgtcacctccatc
Rv0187	Forward	gccgatgttggttaccgctg
	Reverse	agcaaccgcaaccgcgattc
Rv1396c	Forward	gacatgaccgtccccctgaa
	Reverse	atcgtgtcgccgctgtccga
Rv1323	Forward	tcgatggcaacgtccaggtc
	Reverse	gtacgcgcgccagcaacaat
Rv1772	Forward	gaccgggtgtttctcaacga
	Reverse	gtgaacgcgcacgcttggtt
Rv0252	Forward	ttgccggcccgctataccaa
	Reverse	gaccacgacgatctcgcgag
Rv0937c	Forward	gaaggcgatcgaacccgtcc
	Reverse	gagcaccttgtcggcgttgg
Rv1047	Forward	cttccggggctctcagacat
	Reverse	gtccaccaatcctcgctgca
Rv0508	Forward	gatttcccggttgaaagagg
	Reverse	atgaacgagcgcacgacgag
Rv1754c	Forward	gggctggacgttgcaattga
	Reverse	tgttcggcaccggtggtcgt

Supplementary Table 2. The effect of the fragment length of Rv0867c on MtrA binding affinity

Motif Length	KD (M)	Full R^2
40bp	1.16E-07	0.907046
35bp	1.64E-07	0.959218
30bp	1.83E-07	0.919595
28bp	1.63E-07	0.917884

27bp	1.61E-07	0.966976
26bp	1.42E-07	0.934603
25bp	4.40E-06	0.964371
24bp	6.97E-05	0.936076
23bp	5.10E-04	0.988186
22bp	NA	0

frontiers

Supplementary Table 3. Summary of MtrA regulons

Functional category	Gene	MtrA target	Function	Essentiality					Dormancy / resuscitation	Persistence
				in vivo		in vitro				
				(Sassetti and Rubin, 2003)	(Rengarajan et al., 2005)	(Minato et al., 2019)	(DeJesus et al., 2017)	(Griffin et al., 2011)		
DNA replication	*dnaA* *	(Fol et al., 2006; Chatterjee et al., 2018)	Chromosomal replication initiator	-	-	+	+	+	+	-
	dnaB	(Gorla et al., 2018)	DNA helicase	-	-	+	+	+	+	-
	dnaN *	(Purushotham et al., 2015; Gorla et al., 2018)	DNA polymerase III subunit beta	-	-	+	+	+	+	-
	holB	(Chatterjee et al., 2018; Gorla et al., 2018)	Clamp loading	-	-	+	+	+	-	-
DNA repair	*dnaE2*	This study	Trans-lesion synthesis	-	-	-	-	-	+	-

	mpg	This study	Base excision repair	-	-	-	-	-	+	-
	nth	(Gorla et al., 2018)	Base excision repair	-	-	-	-	-	-	-
	ruvA	This study	Homologous recombination	-	-	-	+	-	+	-
	radA	This study	Radiation damage repair	-	-	-	-	-	-	-
	mku	(Gorla et al., 2018)	Non-homologous end joining	-	-	-	-	-	+	-
	ligD	(Gorla et al., 2018)	Non-homologous end joining	-	-	-	-	+	+	-
Cell division	*sepF*	(Gorla et al., 2018)	Early divisome	-	-	+	+	+	-	-
	ftsW	This study	Late divisome	-	-	+	+	+	-	-
	wag31 *	(Plocinska et al., 2012; Minch et al., 2015; Gorla et al., 2018)	Regulation of Cytokinesis	-	-	+	+	+	+	-
	chiZ	This study	Cell wall hydrolase	-	-	-	-	-	-	-
	cwlM	This study	Peptidoglycan hydrolase	-	-	+	+	+	-	-

	ami1	(Gorla et al., 2018)	N-acetylmuramoyl-L-alanine amidase	+	-	+	-	-	-	+
	ripA *	(Plocinska et al., 2012; Minch et al., 2015; Gorla et al., 2018)	Peptidoglycan endopeptidase	-	-	+	+	+	-	+
	rpfB *	(Minch et al., 2015; Sharma et al., 2015; Chatterjee et al., 2018; Gorla et al., 2018)	Resuscitation-promoting factor	-	-	-	-	-	+	-
Cell envelope assembly	*murA*	This study	UDP-N-acetylglucosamine 1-carboxyvinyl transferase	-	-	+	+	+	-	-
	murJ	This study	Peptidoglycan lipid II flippase	+	-	+	+	+	-	-
	ponA2	(Minch et al., 2015; Chatterjee et al., 2018; Gorla et al., 2018)	Bifunctional penicillin-insensitive transglycosylase or transpeptidase	-	+	-	-	-	-	-

ldtA *	(Minch et al., 2015; Chatterjee et al., 2018; Gorla et al., 2018)	L,D-transpeptidase	-	-	-	-	-	-	-
ldtC	(Minch et al., 2015; Gorla et al., 2018)	L,D-transpeptidase	-	-	-	-	-	-	-
PBP-lipo *	(Minch et al., 2015; Gorla et al., 2018)	Penicillin-binding lipoprotein	-	-	-	-	-	-	-
dacB1 *	(Gorla et al., 2018)	Penicillin-binding protein	-	-	-	-	-	-	-
rpfA *	(Minch et al., 2015; Chatterjee et al., 2018; Gorla et al., 2018)	Resuscitation-promoting factor	-	-	-	-	-	+	-
rpfC *	(Minch et al., 2015; Chatterjee et al., 2018;	Resuscitation-promoting factor	-	-	-	-	-	+	+

	Gorla et al., 2018)								
rpfE	(Gorla et al., 2018)	Resuscitation-promoting factor	-	-	-	-	-	+	-
rv2525c *	(Minch et al., 2015; Gorla et al., 2018)	Peptidoglycan glycoside hydrolase	-	-	-	-	-	-	-
rv0950c	(Minch et al., 2015; Chatterjee et al., 2018; Gorla et al., 2018)	Murein DD-endopeptidase and hydrolase activator	+	-	-	-	-	-	-
glf	(Gorla et al., 2018)	UDP-galactopyranose mutase	-	-	+	+	+	-	-
aftC	This study	Alpha-(1->3)-arabinofuranosyltransferase	-	-	+	+	+	-	-
ppgS	This study	Polyprenyl-phospho-N-acetylgalactosaminyl synthase	+	-	-	-	-	-	-
rv3779	This study	Galactosaminyl transferase	-	-	-	-	-	-	-
fas	(Minch et al., 2015;	Fatty acid synthase	-	-	+	+	+	+	-

	Chatterjee et al., 2018; Gorla et al., 2018)								
accD4	This study	Propionyl-CoA carboxylase subunit beta	-	-	+	+	+	+	-
fabH	This study	3-oxoacyl-ACP synthase III	-	-	-	-	-	-	-
fbpA	This study	Diacylglycerol acyltransferase/mycolyltransfe rase	-	-	+	-	+	+	+
fbpB *	(Rajagopal an et al., 2010; Minch et al., 2015; Chatterjee et al., 2018; Gorla et al., 2018)	Diacylglycerol acyltransferase/mycolyltransfe rase	-	-	-	-	-	+	+
fbpC	(Minch et al., 2015; Gorla et al., 2018)	Diacylglycerol acyltransferase/mycolyltransfe rase	-	-	-	-	-	-	+
umaA	(Minch et al., 2015;	Mycolic acid methyltransferase	-	-	-	-	-	+	-

		Gorla et al., 2018)								
	rv2252	This study	Diacylglycerol kinase	-	-	-	-	-	-	-
	pimE	(Minch et al., 2015; Gorla et al., 2018)	Alpha-(1-2)-phosphatidylinositol pentamannoside mannosyltransferase	-	-	-	-	+	-	-
	mptB	This study	Alpha-(1->6)-mannopyranosyltransferase	-	-	+	+	+	-	-
	mptC	This study	Alpha-(1-2)-phosphatidylinositol mannoside mannosyltransferase	-	-	-	-	-	-	-
	capA	This study	Alpha-(1-5)-mannosyltransferase	-	-	-	-	-	-	-
Lipopolysaccharide biosynthesis	*gmhA*	This study	Phosphoheptose isomerase	-	-	-	-	-	-	-
	rv3037c	This study	S-adenosylmethionine-dependent methyltransferase	-	+	-	-	-	-	-
Transcriptional regulators	*sigB*	This study	Regulates stationary phase and general stress resistance	-	-	+	-	-	+	+

sigD *	(Gorla et al., 2018)	Regulates lipid metabolism and cell wall related processes	-	-	-	-	-	+	+
sigE	(Gorla et al., 2018)	Regulates stringent response	-	-	+	-	+	+	+
sigH	This study	Regulates heat shock and oxidative stress response	-	+	-	-	-	+	+
rbpA	This study	Binds to RNA polymerase, stimulating transcription from principal	-	-	+	+	-	+	-
carD	(Minch et al., 2015; Gorla et al., 2018)	RNA polymerase-binding transcription factor	-	-	+	+	+	-	+
whiB1	(Gorla et al., 2018)	Regulates growth, secretion system, dormancy and resuscitation	-	-	+	+	-	+	-
whiB3 *	(Gorla et al., 2018)	Regulates related surface-lipid biosynthesis	-	-	-	-	-	+	+
whiB4 *	(Minch et al., 2015; Gorla et al., 2018)	Regulates oxidative stress response	-	-	-	-	-	-	-

whiB7	This study	Regulates innate multidrug resistance	-	-	-	-	-	-	-
mosR	This study	Regulates hypoxia/intracellular survival	-	-	+	-	+	+	-
mihF	This study	Regulates virulence and housekeeping genes	-	-	-	-	-	-	-
mcdR	(Gorla et al., 2018)	Regulates cell division and DNA repair	-	-	+	+	+	-	-
mtrA	(Minch et al., 2015; Chatterjee et al., 2018)	Regulates DNA replication and cell division.	+	+	+	+	+	-	+
espR	This study	Regulates ESX-1 system	-	-	-	-	-	-	-
rv0177	This study	Regulates cell wall structure	-	+	-	-	-	-	-
rv0822c	(Minch et al., 2015; Chatterjee et al., 2018)	Regulates cell envelope	-	-	-	-	-	-	-
rv1358	This study	Adenylate cyclase	-	-	-	-	-	-	-
rv1404	This study	Regulates acid stress adaptation	-	-	-	-	-	-	-

	rv2618	This study	ArsR family	-	-	-	-	-	-	-
	rv3058c	(Gorla et al., 2018)	TetR family	-	-	-	-	-	-	-
	rv3488	(Gorla et al., 2018)	Regulates metal-detoxifying	-	-	-	-	-	-	-
Signal transduction	*pknA*	This study	Regulates morphological, cell division/differentiation	-	-	+	+	+	-	-
	pknF	This study	Regulates glucose transport, cell growth, and septum formation	-	-	-	-	-	-	-
	pknH	This study	Regulates LAM and AG biosynthesis	-	-	-	-	-	+	-
	rv3354	(Minch et al., 2015; Gorla et al., 2018)	Regulates metalloprotease activity	-	-	-	-	-	-	-
Translation	*leuS*	(Gorla et al., 2018)	Leucine-tRNA ligase	-	-	+	+	+	-	-
	rpsF	This study	30S ribosomal protein S6	-	-	-	+	+	-	-
	rv0208c	(Minch et al., 2015;	tRNA (guanine-N(7)-)-methyltransferase	-	-	-	-	+	-	-

		Gorla et al., 2018)								
	rplB	This study	50S ribosomal protein L2	-	-	+	+	+	+	-
	rpsT	(Gorla et al., 2018)	30S ribosomal protein S20	-	-	-	-	-	-	-
	rv2631	(Gorla et al., 2018)	RNA-splicing ligase	-	-	-	-	-	+	-
	rv2842c	This study	Ribosome maturation factor	-	-	-	-	-	-	-
	rpmB	This study	50S ribosomal protein L28	-	-	-	-	-	-	-
	rv3241c	(Minch et al., 2015; Gorla et al., 2018)	Ribosome-associated translation inhibitor	-	-	-	-	-	-	-
	truA	This study	tRNA pseudouridine synthase A	-	-	-	+	+	-	-
	rpmH	This study	50S ribosomal protein L34	-	-	+	-	-	-	-
	rrl	This study	23S ribosomal RNA	-	-	-	-	-	-	-
Chaperones	*grpE*	(Gorla et al., 2018)	Stress response protein	-	-	+	+	+	+	-

Lipid metabolism	*nrp*	This study	Peptide synthetase	+	-	-	-	-	-	-
	fadE5	(Gorla et al., 2018)	Acyl-CoA dehydrogenase	-	-	-	-	-	+	-
	fadD30	(Minch et al., 2015; Gorla et al., 2018)	Long-chain-fatty-acid--AMP ligase	-	-	-	-	+	+	-
	echA2	(Gorla et al., 2018)	Enoyl-CoA hydratase	-	-	-	-	-	-	-
	rv0856	This study	Chalcone/flavanone-binding protein	-	-	-	-	-	-	-
	rv0857	This study	Chalcone/flavanone-binding protein	-	-	-	-	-	-	-
	rv0947c	This study	Probable mycolyl transferase, pseudogene	-	-	-	-	-	-	-
	rv0974c	(Gorla et al., 2018)	Acetyl-/propionyl-CoA carboxylase subunit beta	-	-	-	-	-	+	-
	pks16	(Gorla et al., 2018)	Polyketide synthase	+	-	-	-	-	-	+
	fadA3	This study	Beta-ketoacyl CoA thiolase	-	-	-	-	-	-	-

fadA4	(Gorla et al., 2018)	Acetyl-CoA acetyltransferase	+	-	-	-	-	-	-
rv1425	(Gorla et al., 2018)	Diacyglycerol O-acyltransferase	-	-	-	-	-	-	-
pks9	This study	Polyketide synthase	-	-	-	-	-	-	-
rv1683	(Gorla et al., 2018)	Bifunctional long-chain acyl-CoA synthase/lipase	-	-	-	-	+	-	-
fadD1	(Gorla et al., 2018)	Fatty-acid--CoA ligase	-	-	-	-	-	-	-
rv1760	This study	Diacylglycerol acyltransferase	-	-	-	-	-	-	-
mbtD	This study	Polyketide synthetase	-	-	-	-	+	+	+
rv2417c	(Gorla et al., 2018)	Degv domain-containing protein	-	-	-	-	-	-	-
echA14	(Gorla et al., 2018)	Enoyl-CoA hydratase	-	-	-	-	-	-	-
fadD9	This study	Fatty-acid--CoA ligase	-	-	-	-	-	-	-
pptT	This study	4'-phosphopantetheinyl transferase	-	-	+	+	+	-	-

	cdsA	This study	Phosphatidate cytidylyltransferase	-	-	+	+	+	-	-
	ppsE	This study	Phthiocerol synthesis polyketide synthase type I	-	-	-	-	-	+	-
	fadD28	This study	Long-chain-fatty-acid--AMP ligase	-	-	-	-	-	-	-
	fadD29	(Minch et al., 2015)	Long-chain-fatty-acid--AMP ligase	-	-	-	-	-	+	+
	rv2958c	(Gorla et al., 2018)	PGL/p-HBAD biosynthesis glycosyltransferase	-	-	-	-	-	-	-
	desA3	(Gorla et al., 2018)	Stearoyl-CoA 9-desaturase	+	-	-	-	+	+	-
	fadD23	(Gorla et al., 2018)	Phthioceranic/hydroxyphthioceranic acid synthase	-	-	-	-	-	-	-
	rv3916c	This study	Acetyltransferase	-	-	-	-	-	-	-
Intermediary metabolism and respiration	*rv0052*	This study	Isonitrile hydratase	-	-	-	-	-	-	-
	rv0063	(Chatterjee et al., 2018; Gorla et al., 2018)	Oxidoreductase	-	-	-	-	-	-	-

htdZ	(Gorla et al., 2018)	3-hydroxyl-thioester dehydratase	-	-	-	-	-	-	-
rv0187	(Gorla et al., 2018)	O-methyltransferase	-	+	-	-	-	-	-
rv0207c	(Gorla et al., 2018)	Putative heme uptake system protein	-	+	-	-	-	-	-
nirB	(Minch et al., 2015; Gorla et al., 2018)	Nitrite reductase large subunit	-	-	-	-	-	+	-
pcp	(Gorla et al., 2018)	Pyrrolidone-carboxylate peptidase	-	-	-	-	-	-	-
rv0365c	(Gorla et al., 2018)	Predicted alpha-1,6-mannanase	-	-	-	-	-	-	-
rv0373c	This study	Carbon monoxyde dehydrogenase large subunit	-	-	-	-	+	-	-
rv0484c	This study	Short-chain type oxidoreductase	-	-	-	-	-	-	-
galE2	This study	UDP-glucose 4-epimerase	-	-	-	-	-	-	-
rv0508	(Gorla et al., 2018)	Glutaredoxin	-	-	-	-	-	-	-

hemD	This study	Uroporphyrin-III C-methyltransferase	-	-	+	+	+	-	-
rv0575c	This study	Oxidoreductase	-	-	-	-	-	+	-
rv0654	(Minch et al., 2015; Gorla et al., 2018)	Carotenoid cleavage oxygenase	-	-	-	-	-	-	-
pepD	This study	Serine protease	-	-	-	-	-	+	+
rv1006	This study	Beta-galactosidase	-	-	-	-	-	-	-
ispE	This study	4-diphosphocytidyl-2C-methyl-D-erythritol kinase	-	-	+	+	+	-	-
rv1105	This study	Possible para-nitrobenzyl esterase (fragment)	-	-	-	-	-	-	-
narG	(Chatterjee et al., 2018; Gorla et al., 2018)	Nitrate reductase subunit alpha	-	-	-	-	-	+	+
mshB	(Minch et al., 2015; Gorla et al., 2018)	1D-myo-inositol 2-acetamido-2-deoxy-alpha-D-glucopyranoside deacetylase	-	-	-	-	+	-	-

lysA	This study	Diaminopimelate decarboxylase	-	-	+	+	+	-	-
rv1320c	This study	Adenylate cyclase	-	-	-	-	-	-	-
rv1360	This study	Oxidoreductase	-	-	-	-	-	-	-
rv1373	(Gorla et al., 2018)	Glycolipid sulfotransferase	-	-	-	-	-	-	-
pyrB	This study	Aspartate carbamoyltransferase	-	-	+	+	+	-	-
gap	(Gorla et al., 2018)	Glyceraldehyde 3-phosphate dehydrogenase	-	-	-	+	+	+	-
hemZ	This study	Ferrochelatase	-	-	+	+	+	+	-
lipL	This study	Esterase	-	-	-	-	-	-	-
gmdA	This study	GDP-D-mannose dehydratase	-	-	-	-	-	-	-
rv1520	This study	Sugar transferase	-	-	-	-	-	-	-
rv1523	(Minch et al., 2015; Chatterjee et al., 2018;	Methyltransferase	-	-	-	-	-	-	-

	Gorla et al., 2018)								
rv1533	This study	Monooxygenase	-	-	-	-	-	-	-
hisD	This study	Histidinol dehydrogenase	-	-	+	+	+	-	-
hisI	This study	Phosphoribosyl-AMP cyclohydrolase	-	-	+	+	+	-	-
rv1700	This study	NUDIX hydrolase	-	-	-	-	-	-	-
mycP5	This study	Membrane-anchored mycosin	-	-	+	+	-	-	-
rv1817	This study	Flavoprotein	-	-	-	-	-	-	-
adhA	This study	Alcohol dehydrogenase A	-	-	-	-	-	-	-
rv1896c	This study	S-adenosyl-L-methionine-dependent methyltransferase	-	-	-	-	-	-	-
aceAa	This study	Isocitrate lyase	-	-	-	-	-	+	-
rv2054	(Gorla et al., 2018)	Dienelactone hydrolase	-	-	-	-	-	-	-
cobH	This study	Precorrin-8X methylmutase	-	-	-	-	-	-	-
cobK	This study	Precorrin-6A reductase	-	-	-	-	-	-	-

cobC	This study	Aminotransferase	+	-	-	-	-	-	-
sseB	This study	Thiosulfate sulfurtransferase	-	-	-	-	-	-	-
plcA	(Gorla et al., 2018)	Membrane-associated phospholipase A	-	-	-	-	-	-	-
rv2435c	This study	Cyclase	-	-	-	-	-	-	-
rv2492	This study	Thymidylate synthase	-	-	-	-	-	-	-
bkdA	This study	3-methyl-2-oxobutanoate dehydrogenase subunit alpha	-	-	-	-	-	+	-
rv2522c	This study	Acetylornithine deacetylase/Succinyl-diaminopimelate desuccinylase or related deacylase	-	-	-	-	-	-	-
rv2542	This study	Alpha/beta hydrolase	-	-	-	-	-	-	-
ribF	This study	Bifunctional riboflavin kinase /FMN adenylyltransferase	-	-	+	+	+	-	-
cysG	This study	Multifunctional uroporphyrin-III C-methyltransferase/precorrin-2 oxidase/ferrochelatase	-	-	+	+	+	+	-

leuC	This study	3-isopropylmalate dehydratase large subunit	-	-	-	+	+	-	-
cyp136	(Gorla et al., 2018)	Cytochrome P450	-	-	-	-	-	-	-
fprA	This study	NADPH-ferredoxin reductase	-	-	-	-	-	-	-
sahH	(Gorla et al., 2018)	Adenosylhomocysteinase	-	-	+	+	+	-	-
lpqC	This study	Esterase	-	-	-	-	-	-	-
moaX	This study	Moad-moae fusion protein	-	-	-	-	-	-	-
nagA	This study	N-acetylglucosamine-6-phosphate deacetylase	-	-	-	-	-	-	-
metC	This study	O-acetylhomoserine sulfhydrylase	-	-	-	-	-	+	-
lytB1	This study	4-hydroxy-3-methylbut-2-enyl diphosphate reductase	-	-	-	-	-	-	-
htdY	This study	3-hydroxyacyl-thioester dehydratase	-	-	-	-	-	-	-
glpK	This study	Glycerol kinase	-	-	-	-	+	-	-

	rv3699	(Gorla et al., 2018)	2-polyprenyl-3-methyl-5-hydroxy-6-metoxy-1,4-benzoquinol methylase	-	-	-	-	-	-	-
	rv3762c	(Gorla et al., 2018)	Hydrolase	-	-	-	-	-	-	-
	rv3777	(Gorla et al., 2018)	Oxidoreductase	-	-	-	-	-	-	-
	rv3787c	(Gorla et al., 2018)	O-Methyltransferase	-	-	-	-	-	-	-
	gltB	This study	Glutamate synthase large subunit	-	-	-	+	+	-	-
	mycP1	(Gorla et al., 2018)	Membrane-anchored mycosin	-	-	-	-	-	-	-
	mycP2	This study	Membrane-anchored mycosin	-	-	-	-	-	-	-
Transporter	*rv0073*	This study	Glutamine ABC transporter ATP-binding protein	-	-	-	-	-	-	-
	mmpL1	(Gorla et al., 2018)	Transmembrane transport protein	-	-	-	-	-	-	+
	mmpS4	(Gorla et al., 2018)	Probable conserved membrane protein	-	-	-	-	-	-	-

mmpL2	(Minch et al., 2015)	Transmembrane transport protein	-	-	-	-	+	-	+
pitA	(Gorla et al., 2018)	Low-affinity inorganic phosphate transporter	-	-	-	-	-	-	-
mmpL5	This study	Transmembrane transport protein	-	-	-	-	-	-	+
pstA1	This study	Phosphate ABC transporter permease	-	+	+	-	-	+	+
rv0986	This study	Adhesion component ABC transporter ATP-binding protein	-	-	-	-	+	-	-
rv1272c	This study	Drug ABC transporter ATP-binding protein	+	-	-	-	-	-	-
mmpL6	This study	Transmembrane transport protein	-	-	-	-	-	-	-
modA	This study	Molybdate ABC transporter substrate-binding lipoprotein	-	-	-	-	-	+	-
mmpL7	This study	Transmembrane transport protein	+	+	-	-	-	-	-
rv3197	This study	ABC transporter ATP-binding protein	-	-	-	-	-	-	-

	rv3680	This study	Anion transporter atpase	-	-	-	-	-	-	-
	mmpL8	(Minch et al., 2015; Gorla et al., 2018)	Integral membrane transport protein	-	-	+	-	+	-	-
Secretion system	*eccC3*	This study	ESX-3 secretion system protein	-	-	+	+	+	-	-
	rv0455c	This study	Siderophore secretion	-	-	+	-	+	-	-
	esxJ	(Gorla et al., 2018)	ESAT-6 like protein	-	-	-	-	-	+	-
	eccD2 *	(Gorla et al., 2018)	ESX-2 secretion system protein	-	-	-	-	-	-	-
Virulence, detoxification, adaptation	*mce1A*	This study	Mce family protein	+	+	-	-	-	-	+
	mce1C	This study	Mce family protein	+	+	-	-	-	-	-
	mce1D	This study	Mce family protein	-	+	-	-	-	-	-
	rv0309	(Minch et al., 2015; Gorla et al., 2018)	L,D-peptidoglycan transpeptidase	-	-	-	-	-	-	-

rv0790c	(Gorla et al., 2018)	Transglutaminase-like enzyme, putative cysteine protease	-	+	-	-	-	-	-
rv1268c	(Minch et al., 2015; Chatterjee et al., 2018; Gorla et al., 2018)	Hypothetical protein	-	-	-	-	-	-	-
ripD	(Gorla et al., 2018)	Cell wall-associated hydrolase	-	-	-	-	-	-	-
katG	(Minch et al., 2015; Gorla et al., 2018)	Catalase-peroxidase	-	-	-	-	+	+	+
mazE5	This study	Antitoxin	-	-	-	-	+	-	-
vapB14	(Gorla et al., 2018)	Antitoxin	-	-	-	-	-	-	-
ripC	(Chatterjee et al., 2018; Gorla et al., 2018)	Endopeptidase	-	-	-	-	+	-	-
mazE8	This study	Antitoxin	-	-	-	-	-	-	-

tyzA	(Gorla et al., 2018)	Acyl-oxazolones synthetase	-	-	-	-	-	-	-
lipP	(Gorla et al., 2018)	Esterase/lipase	-	+	-	-	-	-	-
lipQ	(Gorla et al., 2018)	Carboxylesterase	-	-	-	-	-	-	-
vapB38	(Gorla et al., 2018)	Antitoxin	-	-	-	-	-	-	-
vapB20	This study	Antitoxin	-	-	-	-	-	-	-
rv2663	This study	Ribonuclease toxin	-	-	-	-	-	-	-
vapB21	(Minch et al., 2015; Gorla et al., 2018)	Antitoxin	-	-	-	-	-	+	-
vapB22	This study	Antitoxin	-	-	-	-	-	+	-
cfp6	This study	Low molecular weight protein antigen 6	-	-	-	-	-	-	-
hpx	(Chatterjee et al., 2018; Gorla et al., 2018)	Non-heme haloperoxidase	-	-	-	-	-	-	-

	yrbE4B	(Gorla et al., 2018)	Integral membrane protein	-	-	-	-	-	-	-
	rv3587c	This study	Membrane protein	-	-	+	+	+	-	-
Pe/ppe	*PPE6*	This study	PPE family protein	-	-	-	-	-	-	-
	PE7 *	(Gorla et al., 2018)	PE family protein	-	-	-	-	-	-	-
	PPE15	(Gorla et al., 2018)	PPE family protein	-	-	-	-	-	+	-
	PE_PGRS 19	(Gorla et al., 2018)	PE-PGRS family protein	-	-	-	-	-	-	-
	PE9	(Chatterjee et al., 2018; Gorla et al., 2018)	PE family protein	-	-	-	-	-	-	-
	PPE17	(Chatterjee et al., 2018)	PPE family protein	-	-	-	-	-	+	-
	PPE19	(Minch et al., 2015; Chatterjee et al., 2018; Gorla et al., 2018)	PPE family protein	-	-	-	-	-	-	-

PE_PGRS 25	(Chatterjee et al., 2018; Gorla et al., 2018)	PE-PGRS family protein	-	-	-	-	-	-	-
PPE21	This study	PPE family protein	-	-	-	-	-	-	-
wag22	This study	PE-PGRS family protein	-	-	-	-	-	+	-
PE18	(Gorla et al., 2018)	PE family protein	-	-	-	-	-	-	-
PE19	(Minch et al., 2015; Gorla et al., 2018)	PE family protein	-	-	-	-	-	-	-
PPE29	This study	PPE family protein	-	-	-	-	-	-	-
PPE33	This study	PPE family protein	-	-	-	-	-	-	-
PPE35	(Minch et al., 2015; Chatterjee et al., 2018; Gorla et al., 2018)	PPE family protein	-	-	-	-	+	-	-
PPE37	This study	PPE family protein	-	-	-	-	-	-	+

	PE_PGRS 38	(Minch et al., 2015; Gorla et al., 2018)	PE-PGRS family protein	-	-	-	-	-	-	-
	PPE38	(Minch et al., 2015; Chatterjee et al., 2018; Gorla et al., 2018)	PPE family protein	-	-	-	-	-	-	-
	PPE45	(Gorla et al., 2018)	PPE family protein	-	-	-	-	-	-	-
	PPE53	(Gorla et al., 2018)	PPE family protein	-	-	-	-	-	+	-
	PE_PGRS 50	(Minch et al., 2015; Gorla et al., 2018)	PE-PGRS family protein	-	-	-	-	-	-	-
	PPE64	This study	PPE family protein	-	-	-	-	-	-	-
Other membrane proteins	*rv0188*	(Minch et al., 2015; Gorla et al., 2018)	Transmembrane protein	-	-	-	-	-	+	-
	rv0556	This study	Transmembrane protein	-	-	+	+	+	-	-

	rv0666	This study	Membrane protein	-	-	-	-	-	-	-
	rv0996	(Minch et al., 2015; Gorla et al., 2018)	Transmembrane protein	-	-	-	-	-	-	-
	rv1481	This study	Membrane protein	-	-	+	+	+	-	-
	rv2434c	This study	Transmembrane protein	-	-	-	-	-	-	-
	rv3857c	(Minch et al., 2015; Chatterjee et al., 2018; Gorla et al., 2018)	Membrane protein	-	-	-	-	-	-	-
Mobilome: prophages, transposons	*rv1036c*	(Gorla et al., 2018)	Probable IS1560 transposase (fragment)	-	-	-	-	-	-	-
	rv1047	(Gorla et al., 2018)	Transposase	-	-	-	-	-	+	-
	rv1588c	This study	Partial REP13E12 repeat protein	-	-	-	-	-	-	-
	rv2647	This study	Chromosome segregation protein	-	-	-	-	-	-	-

	rv3431c	This study	Possible transposase (fragment)	-	-	-	-	-	-	-
Non-coding RNA	*ncRv11147c*	This study	Putative small regulatory RNA	-	-	-	-	-	-	-
Unknown	*lprO*	(Minch et al., 2015; Gorla et al., 2018)	Lipoprotein	-	+	-	-	-	-	-
	rv0320	(Minch et al., 2015; Gorla et al., 2018)	Hypothetical protein	-	-	-	-	-	-	-
	rv0446c	This study	Transmembrane protein	-	-	-	-	-	-	-
	rv0787	This study	Hypothetical protein	-	-	-	-	-	-	-
	lpqQ	This study	Lipoprotein	-	-	-	-	-	-	-
	rv1116	(Minch et al., 2015; Chatterjee et al., 2018; Gorla et al., 2018)	Hypothetical protein	-	-	-	-	-	-	-
	rv1697	This study	Hypothetical protein	-	-	+	+	+	-	-

rv1754c	(Minch et al., 2015; Gorla et al., 2018)	Hypothetical protein	-	-	-	-	-	-	-
rv1772	(Minch et al., 2015; Gorla et al., 2018)	Hypothetical protein	-	-	-	-	-	-	-
rv1780	This study	Hypothetical protein	-	-	-	-	-	-	-
rv1815	(Minch et al., 2015; Chatterjee et al., 2018; Gorla et al., 2018)	Hypothetical protein	-	-	-	-	-	-	-
rv1825	This study	Hypothetical protein	-	-	-	-	-	-	-
rv1976c	(Gorla et al., 2018)	Hypothetical protein	-	-	-	-	-	-	-
rv1995	This study	Hypothetical protein	-	-	-	-	-	-	-
rv2342	(Gorla et al., 2018)	Hypothetical protein	-	-	-	-	-	-	-
rv2426c	This study	Moxr-like atpase	-	-	-	-	-	-	-

rv2632c	This study	Hypothetical protein	-	-	-	-	-	-	-
rv2742c	(Gorla et al., 2018)	Hypothetical protein	-	-	-	-	-	-	-
rv3099c	This study	Hypothetical protein	-	-	-	-	-	-	-
rv3209	(Gorla et al., 2018)	Hypothetical protein	-	-	-	-	-	-	-
rv3898c	This study	Hypothetical protein	-	-	-	-	-	-	-

* Verified MtrA target genes.

3 Supplementary References

Chatterjee, A., Sharma, A.K., Mahatha, A.C., Banerjee, S.K., Kumar, M., Saha, S., et al. (2018). Global mapping of MtrA-binding sites links MtrA to regulation of its targets in Mycobacterium tuberculosis. *Microbiology (Reading)* 164(1)**,** 99-110. doi: 10.1099/mic.0.000585.

DeJesus, M.A., Gerrick, E.R., Xu, W., Park, S.W., Long, J.E., Boutte, C.C., et al. (2017). Comprehensive Essentiality Analysis of the Mycobacterium tuberculosis Genome via Saturating Transposon Mutagenesis. *mBio* 8(1). doi: 10.1128/mBio.02133-16.

Fol, M., Chauhan, A., Nair, N.K., Maloney, E., Moomey, M., Jagannath, C., et al. (2006). Modulation of Mycobacterium tuberculosis proliferation by MtrA, an essential two-component response regulator. *Mol Microbiol* 60(3)**,** 643-657. doi: 10.1111/j.1365-2958.2006.05137.x.

Gorla, P., Plocinska, R., Sarva, K., Satsangi, A.T., Pandeeti, E., Donnelly, R., et al. (2018). MtrA Response Regulator Controls Cell Division and Cell Wall Metabolism and Affects Susceptibility of Mycobacteria to the First Line Antituberculosis Drugs. *Front Microbiol* 9**,** 2839. doi: 10.3389/fmicb.2018.02839.

Griffin, J.E., Gawronski, J.D., Dejesus, M.A., Ioerger, T.R., Akerley, B.J., and Sassetti, C.M. (2011). High-resolution phenotypic profiling defines genes essential for mycobacterial growth and cholesterol catabolism. *PLoS Pathog* 7(9)**,** e1002251. doi: 10.1371/journal.ppat.1002251.

Minato, Y., Gohl, D.M., Thiede, J.M., Chacón, J.M., Harcombe, W.R., Maruyama, F., et al. (2019). Genomewide Assessment of Mycobacterium tuberculosis Conditionally Essential Metabolic Pathways. *mSystems* 4(4). doi: 10.1128/mSystems.00070-19.

Minch, K.J., Rustad, T.R., Peterson, E.J., Winkler, J., Reiss, D.J., Ma, S., et al. (2015). The DNA-binding network of Mycobacterium tuberculosis. *Nat Commun* 6**,** 5829. doi: 10.1038/ncomms6829.

Plocinska, R., Purushotham, G., Sarva, K., Vadrevu, I.S., Pandeeti, E.V., Arora, N., et al. (2012). Septal localization of the Mycobacterium tuberculosis MtrB sensor kinase promotes MtrA regulon expression. *J Biol Chem* 287(28)**,** 23887-23899. doi: 10.1074/jbc.M112.346544.

Purushotham, G., Sarva, K.B., Blaszczyk, E., Rajagopalan, M., and Madiraju, M.V. (2015). Mycobacterium tuberculosis oriC sequestration by MtrA response regulator. *Mol Microbiol* 98(3)**,** 586-604. doi: 10.1111/mmi.13144.

Rajagopalan, M., Dziedzic, R., Al Zayer, M., Stankowska, D., Ouimet, M.C., Bastedo, D.P., et al. (2010). Mycobacterium tuberculosis origin of replication and the promoter for immunodominant secreted antigen 85B are the targets of MtrA, the essential response regulator. *J Biol Chem* 285(21)**,** 15816-15827. doi: 10.1074/jbc.M109.040097.

Rengarajan, J., Bloom, B.R., and Rubin, E.J. (2005). Genome-wide requirements for Mycobacterium tuberculosis adaptation and survival in macrophages. *Proc Natl Acad Sci U S A* 102(23)**,** 8327-8332. doi: 10.1073/pnas.0503272102.

Sassetti, C.M., and Rubin, E.J. (2003). Genetic requirements for mycobacterial survival during infection. *Proc Natl Acad Sci U S A* 100(22)**,** 12989-12994. doi: 10.1073/pnas.2134250100.

Sharma, A.K., Chatterjee, A., Gupta, S., Banerjee, R., Mandal, S., Mukhopadhyay, J., et al. (2015). MtrA, an essential response regulator of the MtrAB two-component system, regulates the transcription of resuscitation-promoting factor B of Mycobacterium tuberculosis. *Microbiology (Reading)* 161(6)**,** 1271-1281. doi: 10.1099/mic.0.000087.
